# Ehrlichiosis and Anaplasmosis among Transfusion and Transplant Recipients in the United States

**DOI:** 10.3201/eid2711.211127

**Published:** 2021-11

**Authors:** Sanjida J. Mowla, Naomi A. Drexler, Cara C. Cherry, Pallavi D. Annambholta, Ian T. Kracalik, Sridhar V. Basavaraju

**Affiliations:** Oak Ridge Institute for Science and Education, Oak Ridge, Tennessee, USA (S.J. Mowla);; Centers for Disease Control and Prevention, Atlanta, Georgia, USA (S.J. Mowla, N.A. Drexler, C.C. Cherry, P.D. Annambholta, I.T. Kracalik, S.V. Basavaraju)

**Keywords:** anaplasmosis, bacteria, disease transmission, donor-derived infection, ehrlichiosis, tickborne infections, transfusion, transplants, transplant-transmitted infections, transfusion-transmitted infections, vector-born infections

## Abstract

Ehrlichiosis and anaplasmosis are emerging tickborne diseases that can also be transmitted through blood transfusions or organ transplants. Since 2000, ehrlichiosis and anaplasmosis cases in the United States have increased substantially, resulting in potential risk to transplant and transfusion recipients. We reviewed ehrlichiosis and anaplasmosis cases among blood transfusion and solid organ transplant recipients in the United States from peer-reviewed literature and Centers for Disease Control and Prevention investigations. We identified 132 cases during 1997–2020, 12 transfusion-associated cases and 120 cases in transplant recipients; 8 cases were donor-derived, and in 13 cases illness occurred <1 year after transplant. Disease in the remaining 99 cases occurred ≥1 year after transplant, suggesting donor-derived disease was unlikely. Severe illness or death were reported among 15 transfusion and transplant recipients. Clinicians should be alert for these possible infections among transfusion and transplant recipients to prevent severe complications or death by quickly treating them.

## Abstract 2

Physicians should be aware that these infections are rare but can have severe outcomes.

Ehrlichiosis and anaplasmosis are emerging tickborne diseases caused by *Ehrlichia* and *Anaplasma* spp. obligate intracellular bacteria (*1*,*2*). Tick bites are the primary route of infection, but transmission can also occur through blood transfusion or solid organ transplantation, because these pathogens infect leukocytes and circulate throughout the blood stream (*2*). In the United States, human ehrlichiosis is caused primarily by *Ehrlichia chaffeensis* but can also result from *E. ewingii* or *E. muris eauclairensis* infections (*1*,*3*). Anaplasmosis is caused by *Anaplasma phagocytophilum* (*1*). Although distinct diseases, ehrlichiosis and anaplasmosis share clinical and laboratory features. Early symptoms often include fever, chills, headache, malaise, myalgia, or nausea, and many infections go unrecognized and undiagnosed (*3*–*5*). Laboratory features often include leukopenia, thrombocytopenia, anemia, and elevated hepatic transaminases (*3*–*5*).

Both diseases have incubation periods of 5–14 days from the time of tick transmission, and during early illness infected asymptomatic persons or those with mild illness might be unknowingly accepted as blood donors (*3*,*5*). In general, higher rates of ehrlichiosis and anaplasmosis are reported among adults >40 years of age, and most patients are men (*5*–*7*). Illness onset is most commonly reported during June and July, corresponding to peak tick activity (*3*,*5*). Approximately half of ehrlichiosis and anaplasmosis patients require hospitalization, and 7% require critical care (*3*,*8*). Case-fatality rates are ≈1% for *E. chaffeensis* ehrlichiosis and 0.3% for anaplasmosis patients based on national surveillance reports (*3*,*5*). In part because of immunosuppressive therapies to prevent organ rejection, transplant and transfusion recipients may be more susceptible to ehrlichiosis and anaplasmosis. Relative risk (RR) for severe outcomes among immunosuppressed compared with immunocompetent case-patients was higher for hospitalization (RR 1.4), life-threatening complications (RR 2.4), and death (RR 2.3), highlighting the potential severity of disease in immunocompromised populations (*3*,*9*–*15*).

In the United States, ehrlichiosis was first reported in 1987 and anaplasmosis in 1994, and both became nationally notifiable diseases in 1999 (*3*,*5*,*16*,*17*). Since 2000, reported cases of ehrlichiosis and anaplasmosis in the United States have increased substantially. Reported *E. chaffeensis* ehrlichiosis cases have increased >10-fold, from 200 in 2000 to 2,093 in 2019 (*18*,*19*). Reported anaplasmosis cases increased >16-fold, from 348 cases in 2000 to 5,655 in 2019 (*19*,*20*).

Increasing rates of reported ehrlichiosis and anaplasmosis might be related to several factors, including improved diagnostics, changes in reporting practices, and expanded human contact with animal reservoirs and tick vectors (*2*,*3*,*21*,*22*). *E. chaffeensis* and *E. ewingii* are primarily transmitted by the lone star tick (*Amblyomma americanum*); *A. phagocytophilum* is transmitted by either the blacklegged tick (*Ixodes scapularis*) or the western blacklegged tick (*I. pacificus*) (*3*,*5*). *E. muris eauclaurensis* is transmitted by *I. scapularis* ticks. *E. chaffeensis* ehrlichiosis is most frequently reported in the southeastern and south-central regions of the United States, and anaplasmosis is most often reported in the upper midwestern and northeastern regions. 

PCR and serologic testing using an indirect immunofluorescence antibody assay are the primary laboratory methods for diagnosing ehrlichiosis and anaplasmosis (*3*,*5*). Because infection transmitted through blood or organs is rare, it might not be diagnosed in solid organ transplant and transfusion recipients. In addition, nonspecific signs and symptoms and a higher index of suspicion for other opportunistic infections might complicate diagnosis (*14*), which is unfortunate because early detection and treatment can prevent severe illness and death (*23*). Here, we summarize and discuss the risks of ehrlichiosis and anaplasmosis cases in the United States among solid organ transplant and transfusion recipients, with a focus on donor-derived infections.

## Methods

We conducted a literature search to identify articles published during January–August 2020 describing ehrlichiosis and anaplasmosis in solid organ transplant or blood transfusion recipients in the United States. We used PubMed search terms “ehrlichiosis AND transfusion,” “ehrlichiosis AND transplant,” “anaplasmosis AND transfusion,” and “anaplasmosis AND transplant.” We included articles describing case reports, case series, or other clinical descriptions related to *Ehrlichia* and *Anaplasma* spp. infections in the setting of solid organ transplantation or transfusion of blood products in the United States. We excluded infections in hematopoietic stem cell recipients because these are not reported to a national notifiable disease system. For articles meeting inclusion criteria, we further reviewed references to identify any case reports or case descriptions not found in the initial PubMed search. Duplicate cases were only counted once for this review. We summarized transplant- and transfusion-associated cases as presented in the literature; we made no additional exclusions based on diagnostic criteria, and we only determined cases to be donor-derived if the authors presented them as such in the literature or investigations.

**Figure 1:**
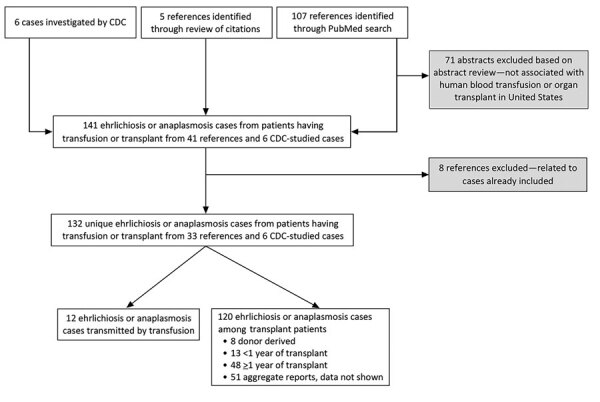
Flow diagram of article and report inclusion for review of ehrlichiosis and anaplasmosis in transfusion and transplant recipients in the United States, 1997–2020. CDC, Centers for Disease Control and Prevention.

In the United States, all suspected or confirmed cases of donor-derived diseases are reported to the Organ Procurement and Transplantation Network and are investigated by the Disease Transmission Advisory Committee (DTAC). Nationally notifiable diseases such as ehrlichiosis and anaplasmosis are referred to the Centers for Disease Control and Prevention (CDC) for investigation and determination of whether the infection was transmitted from a donor to a recipient. We also included cases of transplant-associated ehrlichiosis and anaplasmosis identified as part of these DTAC investigations by CDC if not already accounted for in the peer-reviewed literature (Figure 1). From each reviewed article or CDC-led case investigation, we collected available information on geographic region, recipient characteristics, *Ehrlichia* or *Anaplasma* species, transmission route, age of blood component transfused or type of organ transplanted, time between transplantation and infection, symptoms and clinical details, diagnostic methods, donor and recipient histories of tick exposure, donor characteristics, likely source of infection, and whether the recipient survived or died.

## Results

### Ehrlichiosis and Anaplasmosis Cases Reported among Transfusion Recipients

**Figure 2:**
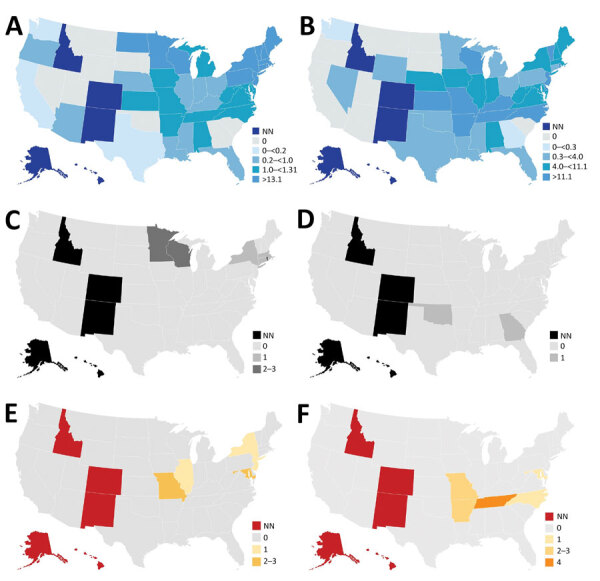
Incidence of ehrlichiosis and anaplasmosis in the United States, 2019, and of cases among transfusion and transplant recipients, 1997–2020. A) Incidence of anaplasmosis per 1 million US residents in 2019. B) Incidence of ehrlichiosis per 1 million US residents in 2019. C) Transfusion-transmitted anaplasmosis cases by recipient state of residence. D) Transfusion-transmitted ehrlichiosis cases by recipient state of residence. E) Organ donor–derived ehrlichiosis cases by recipient state of residence. We identified no organ donor–derived anaplasmosis cases. F) Organ donor–derived ehrlichiosis cases with onset <1 year after transplant by recipient state of residence. We identified 2 additional donor-derived ehrlichiosis cases with onset <1 year after transplant; however, recipient state of residence was unknown. NN, not notifiable.

During the 1997–2020 investigation period, 12 cases of transfusion-transmitted ehrlichiosis or anaplasmosis were reported in the United States (Appendix Table 1). Of the 12 transfusion-transmitted cases, 8 resulted from transfused red blood cell components and 3 from transfused platelet components (2 apheresis and 1 whole blood–derived); the component for 1 case was not identified. Ten (83.3%) of 12 transfusion-associated cases were *A. phagocytophilum* infections; 1 case was associated with *E. ewingii* and 1 with *E. chaffeensis* (Figure 2, panels C, D). Median age of transfusion recipients was 66 years (range 9–85 years); sex was equally distributed. Of the transfusion-associated cases of ehrlichiosis and anaplasmosis, 3 occurred in Minnesota; 2 in Wisconsin; and 1 each in Georgia, Rhode Island, Connecticut, Massachusetts, New York, and Oklahoma (Figure 2, panels A, B). Disease in all cases was diagnosed using PCR, and additional serologic testing was used for 2 cases. Most (83.3%) transfusion case-patients survived infection; one third of cases were associated with mild symptoms. Intensive care unit (ICU) treatment was noted for 2 anaplasmosis patients, prompted by respiratory failure, hypotension, and hypoxia. In addition, 3 anaplasmosis patients had multisystem organ failure, but ICU treatment was not mentioned for these cases. Two patients died, one from anaplasmosis and the other from other medical complications.

### Ehrlichiosis and Anaplasmosis Cases Reported among Solid Organ Transplant Recipients

During the investigation period, 107 cases of ehrlichiosis and 7 cases of anaplasmosis were described in the literature among solid organ transplant recipients. Of these, 63 included patient-specific information. We described the remaining 51 cases in aggregate and reported on them separately. An additional 6 cases of ehrlichiosis among solid organ transplant recipients were investigated by CDC, resulting in a total of 120 cases of ehrlichiosis or anaplasmosis described among transplant recipients during the investigation period.

*E. chaffeensis* ehrlichiosis was the most common organ donor-derived infection, reported among 8 solid organ transplant recipients: 2 cases we identified from the literature and the 6 investigated by CDC (Appendix Table 2). Of these, 7 (87.5%) case-patients were kidney transplant recipients and 1 was a liver transplant recipient. Fever was reported among all solid organ transplant recipients. Diagnostic methods were PCR (87.5%) and serologic testing (12.5%). Most (87.5%) patients were male, median age was 57 years (range 5–69 years), and median time between transplantation and infection was 13.5 days (range 10–25 days). Of the donor-derived ehrlichiosis cases, 2 occurred in Maryland; 3 in Missouri; and 1 each in New York, New Jersey, and Illinois (Figure 2, panel E). Among the 8 donor-derived ehrlichiosis cases, 2 deaths were reported among the kidney transplant recipients, both occurring <1 month after transplantation (Appendix Table 3). None of the confirmed transplant-derived case reports described ICU admission among the patients.

Thirteen cases of ehrlichiosis and anaplasmosis occurred <1 year after transplantation but could not be confirmed as donor-derived. The median time between transplantation and symptom onset in these 13 cases was 5 months (range 2 weeks–9 months) (Appendix Table 4). Of those cases, 4 were identified in Tennessee (Figure 2, panel F). *E. chaffeensis* infection was associated with 11 (84.6%) cases, and *A. phagocytophilum* infection was associated with 2 (15.4%) cases. Of the 13 cases, 5 (38.5%) occurred in kidney recipients, 4 (30.8%) in heart recipients, 2 (15.4%) in kidney and pancreas recipients, and 1 (7.7%) in a liver and lung recipient. Most (76.9%) patients were male, median age was 50 years (range 35–63 years), and PCR was the most common (76.9%) diagnostic method. Most (92.3%) patients infected <1 year after transplantation survived; however, 1 kidney and pancreas recipient with *A. phagocytophilum* infection died.

Forty-eight records of individual cases of ehrlichiosis or anaplasmosis that occurred ≥1 year after transplant were most likely attributable to posttransplant infections acquired through tick bites (Appendix Table 5). Median time from transplant to symptom onset was 6 years (range 1–21 years), most patients (75.0%) were male, and median age was 51 years (range 11–73 years). Among these patients, records described *Ehrlichia* infection for 43 (89.6%) and *Anaplasma* infection for 5 (10.4%). *E. chaffeensis* was implicated in 30 (69.8%) of 43 cases of ehrlichiosis, *E. ewingii* in 12 (27.9%), and an unspecified *Ehrlichia* sp. in 1 (2.3%). Twenty-three patients (53.5%) received kidneys, 8 (18.6%) livers, 7 (16.3%) lungs, 4 (9.3%) hearts, and 1 (2.3%) a pancreas. Of the 5 reported cases of anaplasmosis occurring ≥1 year after transplant, 4 (80.0%) occurred in kidney recipients and 1 (20.0%) in a liver recipient. Among the 43 cases of ehrlichiosis in solid organ transplant recipients who had illness onset ≥1 year after transplant, 26 (60.6%) occurred in Missouri and 12 (27.9%) in Tennessee; exact location was not specified in 1 report, but the case occurred in Alabama, Tennessee, or Mississippi. Among the 5 cases of anaplasmosis, 2 occurred in Minnesota, 1 in Maine, 1 in Wisconsin, and 1 in Connecticut. One patient had possible reactivation of a previous *Anaplasma* infection secondary to potent immunosuppression 9 months after the original infection. There were 2 reports of *Ehrlichia* reinfection that described distinct strains of *E. chaffeensis* and *E. ewingii* found in a secondary infection. Secondary hemophagocytic lymphohistiocytosis, which is characterized by severe and potentially fatal inflammation, developed in 1 kidney transplant recipient. Of the solid organ transplant recipients with ehrlichiosis or anaplasmosis occurring ≥1 year after transplant, 47 (97.9%) of 48 survived. One pancreas recipient with an *E. chaffeensis* infection died. Seven cases occurring ≥1 year of solid organ transplantation did report ICU admission, possibly relating to long-term immunosuppression among transplant recipients.

Of the 51 cases of ehrlichiosis from Missouri for which we report data in aggregate, 18 (35.3%) occurred in kidney recipients, 12 (23.5%) in heart recipients, 12 (23.5%) in lung recipients, 7 (13.7%) in liver recipients, and 2 (4.0%) in kidney-pancreas recipients. Additional information on disease, pathogen species, and demographics was not available for these 51 cases.

## Discussion

This study reviewed ehrlichiosis and anaplasmosis cases among transfusion and solid organ transplant recipients described in published literature and reports from 6 CDC investigations. During the study period, 12 cases of transfusion-transmitted ehrlichiosis or anaplasmosis were reported, 2 of which resulted in death. In addition, 8 cases of organ donor–derived ehrlichiosis were reported (7 kidney transplant recipients and 1 liver recipient), 2 of which resulted in death. *A. phagocytophilum* was the most common causative agent among transfusion-derived infections. In contrast, among transplant recipients, *E. chaffeensis* was the most common causative agent. Donor-derived infections were observed among patients of a broad range of ages (5–85 years). Although children are not considered a high-risk group for anaplasmosis in particular, pediatric infection should not be discounted among transfusion and transplant recipients. Time from transplant or transfusion to the development of signs and symptoms varied widely and in most cases was longer than the typical incubation period for a tick-transmitted infection. Delays in symptom onset might be affected by the colony size and the site of the inoculated bacteria. Transfusion- or transplant-transmitted ehrlichiosis and anaplasmosis are rare but can result in severe outcomes including the death of the recipient.

Although known cases of transfusion- and transplant-transmitted ehrlichiosis or anaplasmosis are uncommon, studies of asymptomatic infection among blood donors and the survivability of infection in blood suggest the risk of transmission is greater than previously recognized. In endemic areas, seroprevalence studies found 11.3% of blood donors in New York, 0.5% in Wisconsin, and 3.5% in Connecticut had detectable antibodies against *A. phagocytophilum* (*24*,*25*). In Iowa, ≈1% of blood donors studied were seropositive for and displayed symptoms of ehrlichiosis after blood donation, although recipient lookback reported by physicians indicated that these transfusions did not result in transfusion-transmitted illnesses (*26*). However, serosurveys do not report active or incident infections, only the proportion of participants previously exposed to an *Ehrlichia* or *Anaplasma* agent. Some studies also examined the survivability of *Ehrlichia* and *Anaplasma* species in donor blood. Blood from anaplasmosis patients in 2 studies found viable *A. phagocytophilum* survived under refrigerated storage conditions for up to 18 days in whole blood (*27*,*28*). One in vitro study reported *E. chaffeensis* remained viable for up to 11 days within refrigerated packed red blood cells (*29*).

Donor-derived ehrlichiosis and anaplasmosis highlight the importance of donor risk mitigation strategies in the setting of blood transfusion and solid organ transplantation and the potential role of laboratory-based screening. Recognizing and diagnosing tickborne diseases is complicated by long incubation periods and potential asymptomatic or mildly symptomatic infections. Although several laboratory tests are used to diagnose ehrlichiosis and anaplasmosis, no tests have been approved by the US Food and Drug Administration (FDA) to screen blood or organ donors for these diseases. Furthermore, serologic screening of donors might not identify active *Ehrlichia* or *Anaplasma* infections or could exclude healthy donors (*28*). PCR testing would more accurately screen contaminated blood and organ products, but no FDA-licensed test is available. Donor deferral on the basis of travel or residence would be impractically broad because of the widespread endemicity of ehrlichiosis and anaplasmosis across regions of the United States. To minimize blood supply disruptions based on travel deferrals for Zika virus and babesia, FDA previously recommended universal antibody testing and regional nucleic acid testing in states with the highest rates of risk for infection (*30*,*31*). Similar screening may eventually be necessary for ehrlichiosis and anaplasmosis as the prevalence and incidence of these infections increases in the United States. CDC will continue to monitor the occurrence of transfusion- or transplant-transmitted ehrlichiosis and anaplasmosis.

Among this study’s limitations, for sources identified through the literature we were limited to the information provided in the case reports, which might not always have represented a full account of the patient experience. Data on donor demographics were extremely limited. Donor state of residence might have provided a more accurate insight on likelihood of donor infection by state which might have broader implications for blood and organ screening criteria. Next, for the purposes of this evaluation, we relied on the characterizations by the original authors or investigators to categorize cases as donor- or transplant-derived ehrlichiosis or anaplasmosis. Finally, we included only cases published in peer-reviewed literature or reported to CDC, possibly underreporting transfusion- and transplant-associated infections. In 2019, the Rickettsial Zoonoses Branch, Division of Vector-Borne Diseases, National Center for Emerging and Zoonotic Infectious Diseases, CDC, added questions relating to recent transfusion and organ transplant to their tickborne rickettsial disease surveillance system (https://www.cdc.gov/ticks/forms/Tick_TBRD_FILL_508.pdf) to better track donor-derived infections.

Because *Ehrlichia* and *Anaplasma* species primarily infect leukocytes, leukoreduction has been presumed to reduce risk for ehrlichiosis and anaplasmosis through passive removal (*23*,*32*,*33*). However, 83% (10/12) of components implicated in transfusion-transmitted cases in this study were leukoreduced. In cell culture models, because of their presence in plasma also, *E. chaffeensis* survived in red blood cells stored in additive solution, suggesting leukoreduction alone will not prevent transmission of *Ehrlichia* and *Anaplasma* species (*29*). Adopting pathogen reduction technology, such as psoralen and ultraviolet light to inactivate infectious agents, for platelet and plasma products would provide additional safety measures to reduce risk for transfusion-transmitted *Ehrlichia* and *Anaplasma* infections. Pathogen-reduced plasma has demonstrated a ≥3.6-log reduction in viable *A. phagocytophilum* (*34*). However, this method has not been approved for red blood cell products, which were implicated in 8 of 12 cases of transfusion-transmitted ehrlichiosis and anaplasmosis in this study.

Identifying risk factors for *Ehrlichia* and *Anaplasma* infections among deceased organ donors is challenging because clinical, demographic, and social information about deceased donors is often obtained from family members, who might not have access to or recall all historical details (*35*). Also, routine laboratory screening of organ donors, required for multiple infectious diseases, including HIV/AIDS, does not yet include testing for infection with *Ehrlichia* and *Anaplasma* species. In addition, the number of posttransplant infections reported in this review highlights the risk among blood product or organ recipients. Therefore, clinicians must closely monitor recipients of blood transfusions or solid organs during long-term management and consider these pathogens when recipients develop signs or symptoms of infection. Because the prevalence of tickborne disease infections is rising, additional risk mitigation interventions will likely be necessary to enhance blood and organ safety.

Appendix Additional information on ehrlichiosis and anaplasmosis among transfusion and transplant recipients in the United States.
