# Supplementary material for: Ehrlichiosis and Anaplasmosis among Transfusion and Transplant Recipients in the United States
Source: Emerg Infect Dis. 2021 Nov;27(11):2768–75. doi: 10.3201/eid2711.211127 (PMC8544963; doi:10.3201/eid2711.211127)
Supplement: Appendix — Additional information on ehrlichiosis and anaplasmosis among transfusion and transplant recipients in the United States. [file 21-1127-Techapp-s1.pdf]

Article DOI: https://doi.org/10.3201/eid2711.211127

Ehrlichiosis and Anaplasmosis among Transfusion and Transplant Recipients in the United States

Appendix

Table 1. Characteristics of transfusion-transmitted ehrlichiosis and anaplasmosis described in the published literature

Author, year	Recipient US state of residence	Donor US state of residence	Recipient sex/age, y	Donor age, y	Genus and species†	Transmission route	Age of organ	Symptoms/clinical details	Method of diagnosis	Recipient outcome
Eastlund, 1999	Minnesota	NA	M/75	NA	*A. phagocytophilum*	Non–LR RBC transfusion	30 d	Fever, rigors, nausea, vomiting	PCR, serology	Survived
Kemperman, 2008	Minnesota	Minnesota	M/68	64	*A. phagocytophilum*	Non–LR RBC transfusion	16 d	Thrombocytopenia, sepsis, multisystem organ failure, hypotension and fever	PCR	Survived
Bachowski, 2008	Minnesota	Minnesota	F/85	81	*A. phagocytophilum*	LR RBC	29 d	NA	PCR	Unknown
Annen, 2012	Wisconsin	Wisconsin	F/81	53	*A. phagocytophilum*	LR RBC transfusion	NA	Fever, myalgias, and pancytopenia, multiorgan failure,	PCR	Survived
Annen, 2012	Wisconsin	Wisconsin	F/51	NA	*A. phagocytophilum*	LR IR RBC transfusion	NA	Fever, fatigue, tachycardia, leukopenia, thrombocytopenia	PCR	Survived
Regan, 2013	Georgia	Florida	M/9	NA	*E. ewingii*	LR IR apheresis platelet transfusion	5 d	Fever, fatigue, malaise, vomiting, diarrhea, and petechial rash, neutropenia, thrombocytopenia, and elevated liver enzymes	PCR	Survived
Alhumaiden, 2013	Rhode Island	Rhode Island	M/64	81	*A. phagocytophilum*	LR RBC transfusion	12 d	Headache, fever, and chills, dyspnea, dry cough, leukopenia, transferred to ICU for respiratory failure	PCR	Survived
Townsend, 2014	Connecticut	New York	M/41	53	*A. phagocytophilum*	LR apheresis platelet transfusion	5 d	NA	PCR	Died from other complications
Shields, 2015	Massachusetts	Massachusetts or New Hampshire	F/34	NA	*A. phagocytophilum*	LR IR RBC transfusion	9 d	Nausea, fevers, myalgia, leukopenia, thrombocytopenia	PCR	Survived
Fine, 2015	Rhode Island	NA	F/78	19	*A. phagocytophilum*	LR WBD platelet transfusion	5 d	Chills, fever, cough, leukopenia, thrombocytopenia hypotension, and hypoxia, transferred to ICU	PCR, serology	Survived
Goel, 2018	New York	New York	M/78	NA	*A. phagocytophilum*	LR IR RBC transfusion	22 d	Fever, tachycardia and hypotension, dyspnea, hypoxia, thrombocytopenia, leukopenia, and transaminitis, multiple organ failure	PCR	Died
Mah, 2018	Oklahoma	NA	F/59	NA	*E. chaffeensis*	LR RBCs and platelets	NA	Fever, pneumonitis, myocarditis, transaminitis, acute kidney injury, graft loss	PCR	Survived

*HLH, hemophagocytic lymphohistiocytosis; ICU, intensive care unit; IHC, immunohistochemistry; IR, infrared; LR, leukoreduced; NA, not available; RBC, red blood cells; SIRS, systemic inflammatory response syndrome
†Genera: *A., Anaplasma*; *E., Ehrlichia*

Table 2. Characteristics of donor-derived ehrlichiosis and anaplasmosis related to solid organ transplant recipients

Author, year†	Recipient US state of residence	Donor US state of residence	Recipient sex/age, y	Donor age, y	Organ	Donor characteristics	Genus and species*	Time, transplant to symptoms	Symptoms/clinical details	Method of diagnosis	Recipient outcome
Sachdev, 2014	Maryland	Maryland	F/57	54	Kidney	Deceased	*E. chaffeensis*	22 d	Fever, pancytopenia, pleural effusion, renal dysfunction	PCR	Survived
Sachdev, 2014	Maryland	Maryland	M/56	54	Kidney	Deceased	*E. chaffeensis*	25 d	Fever, lethargy, thrombocytopenia, anemia, features of TTP-HUS, acute anuric kidney failure	Serology	Survived
CDC†, 2019	Missouri	Kansas	M/17	38	Kidney	Living	*E. chaffeensis*	16 d	Fever	PCR	Survived
CDC†, 2020	New Jersey	Delaware	M/70	57	Kidney	Deceased	*E. chaffeensis*	18 d	Fever, intermittent diarrhea, chills, mild cough, fatigue, chronic bilateral, lower extremity mild swelling (Right side greater than left), pancytopenia	PCR	Died
CDC†, 2020	New York	Delaware	M/66	57	Kidney	Deceased	*E. chaffeensis*	11 d	Fever, re-hospitalized and subsequently developed progressive multisystem organ failure (transplant kidney, liver, lung, brain) in the setting of pancytopenia	PCR	Died
CDC†, 2020	Missouri	Missouri	M/5	29	Kidney	Deceased	*E. chaffeensis*	10 d	Fever, pancytopenia	PCR	Survived
CDC†, 2020	Missouri	Missouri	M/5	29	Kidney	Deceased	*E. chaffeensis*	11 d	Fever, hyponatremia, dehydrations,	PCR	Survived
CDC†, 2020	Illinois	Missouri	M/69	29	Liver	Deceased	*E. chaffeensis*	11 d	Fever, headache, malaise, agitation	PCR	Survived

*Genus: *Ehrlichia*
†Centers for Disease Control and Prevention–led investigation

Table 3. Characteristics of ehrlichiosis and anaplasmosis related deaths among solid organ transplant recipients

Author, year†	Recipient US state of residence	Donor US state of residence	Recipient sex/age, y	Donor age, y	Organ	Genus and species†	Time, transplant to symptoms	Symptoms/clinical details	Method of diagnosis	Likely source of infection
Trofe, 2001	Southeastern United States	NA	M/50	NA	Kidney, Pancreas	*A. phagocytophilum*	9 mo	Weight loss, low-grade fever, and leukopenia, increases in LDH and serum creatinine	Bone marrow biopsies and peripheral blood smears	Reactivation of *Anaplasma* secondary to potent immunosuppression or possible tick exposure
Trofe, 2001	Southeastern United States	NA	F/ 41	NA	Pancreas	*E. chaffeensis*	20 mo	Allograft rejection, Fever, leukopenia, thrombocytopenia, increases in LDH and serum creatinine	Bone marrow biopsies and peripheral blood smears	Reactivation of *Ehrlichia* secondary to potent immunosuppression or possible tick exposure
CDC‡, 2020	New York	Delaware	M/66	57	Kidney	*E. chaffeensis*	11 d	Fever, re-hospitalized and subsequently developed progressive multisystem organ failure (transplant kidney, liver, lung, brain) in the setting of pancytopenia	PCR	Donor-derived
CDC‡, 2020	New Jersey	Delaware	M/70	57	Kidney	*E. chaffeensis*	18 d	Fever, intermittent diarrhea, chills, mild cough, fatigue, chronic bilateral, lower extremity mild swelling (Right side greater than left), pancytopenia	PCR	Donor-derived

Table 4. Characteristics of ehrlichiosis and anaplasmosis cases occurring within 1 year of solid organ transplantation

Author, year	Recipient state of US residence	Sex/Age, y	Organ	Genus and species†	Time, transplant to symptoms	Symptoms/clinical details	Method of diagnosis	Tick exposure	Donor characteristics	Likely source of infection	Recipient outcome
Schutze, 1997	Arkansas	NA	Kidney	*E. chaffeensis*	6 wk	Fever	Serology; morulae seen on bone marrow	NA	Living, related	NA	Survived
Tan, 2001	Maryland	M/47	Liver	*E. chaffeensis*	8 mo	Hypotension, fever, and tachypnea, thrombocytopenia, acute cellular rejection, progressive multi-system organ failure, transferred to ICU	PCR	Yes	NA	Post-transplant infection; removed tick from skin 10 d before admission	Survived
Trofe, 2001	Alabama, Tennessee, or Mississippi	M/50	Kidney-Pancreas	*A. phagocytophilum*	9 mo	Weight loss, low-grade fever, leukopenia, rejection of the kidney and pancreas most likely due to chemotherapy	Peripheral blood smears	NA	NA	Living in heavily wooded area or reactivation secondary to potent immunosuppression	Died
Trofe, 2001	Alabama, Tennessee, or Mississippi	M/38	Kidney-Pancreas	*A. phagocytophilum*	2 mo	Fever, leukopenia, hyperglycemia, and elevated LDH, severe rejection of pancreas most likely unrelated to anaplasmosis	Bone marrow biopsy	NA	NA	Living in heavily wooded area or reactivation secondary to potent immunosuppression	Survived
Cotant, 2006	Tennessee	M/60	Kidney	*E. chaffeensis*	6 mo	Fever, weakness, leukopenia, thrombocytopenia, transferred to ICU	PCR; IHC of bone marrow	No	Deceased	Post-transplant infection	Survived
Thomas, 2007	Tennessee	M/59	Heart	*E. chaffeensis*	113 d	Fever, nausea	PCR	NA	NA	NA	Survived
Thomas, 2007	Tennessee	M/38	Heart	*E. chaffeensis*	269 d	Fever, headache, nausea, vomiting	PCR	NA	NA	NA	Survived
Thomas, 2007	Tennessee	M/54	Heart	*E. chaffeensis*	203 d	Fever, headache, nausea, vomiting, diarrhea, cough	PCR	NA	NA	NA	Survived
Lawrence, 2009	Missouri	F/58	Lung	*E. chaffeensis*	9 mo	Fever, headache	PCR	NA	NA	NA	Survived
Lawrence, 2009	Missouri	M/44	Kidney	*E. chaffeensis*	1 mo	Fever	PCR	NA	NA	NA	Survived
Lawrence, 2009	Missouri	M/63	Heart	*E. chaffeensis*	8 mo	Fever, malaise	PCR	NA	NA	NA	Survived
Masterson, 2020	North Carolina	F/35	Kidney	*E. chaffeensis*	4 mo	Fever, headache, leukopenia, thrombocytopenia	PCR	No	Deceased	Post-transplant infection	Survived
Hassan, 2020	Arkansas	M/57	Kidney	*E. chaffeensis*	2 wk	Fever, rigors, sweats, nausea, fatigue, headache, leukopenia, thrombocytopenia, transaminitis, renal dysfunction, transferred to ICU	PCR	No	Deceased	Post-transplant infection	Survived

*HLH, hemophagocytic lymphohistiocytosis; ICU, intensive care unit; IHC, immunohistochemistry; LDH, lactate dehydrogenase; NA, not available; SIRS, systemic inflammatory response syndrome
†Genera: *A., Anaplasma*; *E., Ehrlichia*

Table 5. Characteristics of ehrlichiosis and anaplasmosis cases occurring ≥1 year of solid organ transplantation*

Author, year†	Recipient state of US residence	Sex/Age, y	Organ	Genus and species‡	Time, transplant to symptoms	Symptoms/clinical details	Method of diagnosis	Tick exposure	Donor characteristics	Likely source of infection	Recipient outcome
Antony, 1995	Kentucky	M/51	Liver	*E. chaffeensis*	6 y	Fever, leukopenia, thrombocytopenia, hypotension, monitored in ICU before therapy	Serology	Y	NA	Post-transplant infection; tick bite 2 wk before symptoms	Survived
Adachi, 1997	Minnesota	M/67	Kidney	*A. phagocytophilum*	16 mo	Fever, rigors, nausea, and myalgias, thrombocytopenia, leukopenia and anemia, hypoxemia	Peripheral smear	Y	Living	Post-transplant infection; tick bite 1 wk before symptoms	Survived
Sadikot, 1999	Arkansas	M/35	Kidney	*E. chaffeensis*	4 y	Fever, headaches, myalgia, pancytopenia, oliguric acute renal failure, transferred to ICU	PCR	Y	Deceased	Post-transplant infection, exposure to ticks 1 wk before symptoms	Survived
Buller, 1999	Missouri	M/11	Kidney	*E. ewingii*	9 y	Fever, headache, myalgia, thrombocytopenia, lymphadenopathy	PCR	Y	Living, related	Post-transplant infection mo prior	Survived
Trofe, 2001	Alabama, Tennessee, or Mississippi	F/41	Pancreas	*E. chaffeensis*	20 mo	Fever, acute respiratory decompensation, multi-system organ failure most likely unrelated to ehrlichiosis, transferred to ICU	Peripheral blood smears	NA	NA	Living in heavily wooded area; possible reactivation secondary to potent immunosuppression	Died
Vannorsdall, 2002	Maine	F/66	Kidney	*A. phagocytophilum*	21 y	Fever, thrombocytopenia, leukopenia	PCR, positive blood smear	N	Deceased	Post-transplant infection	Survived
Safdar, 2002	Missouri	M/38	Lung	*E. chaffeensis*	2 y	Fever, myalgia, headache, acute renal failure, developed features of thrombotic thrombocytopenic purpura	PCR	N	NA	Post-transplant infection	Survived
Liddell, 2002	Missouri	M/56	Liver	*E. chaffeensis*	5 y	Fever, myalgias, headache, nausea, leukopenia, thrombocytopenia	Serology	Y	NA	Post-transplant infection (reinfection)	Survived
Liddell, 2002	Missouri	M/58	Liver	*E. chaffeensis*	7 y	Fever, headache, arthralgias, leukopenia, thrombocytopenia	PCR	Y	NA	Post-transplant infection (reinfection)	Survived
Thomas, 2007	Tennessee	M/50	Kidney	*E. chaffeensis*	3.9 y	Fever, headache	Serology	NA	NA	NA	Survived
Thomas, 2007	Tennessee	F/34	Kidney	*E. ewingii*	3.8 y	Fever, headache, myalgia	PCR	NA	NA	NA	Survived
Thomas, 2007	Tennessee	F/15	Kidney	*E. ewingii*	5.3 y	Fever, cough, nausea, vomiting	PCR	NA	NA	NA	Survived
Thomas, 2007	Tennessee	M/17	Liver	*E. chaffeensis*	16.4 y	Fever, headache, anorexia	PCR	NA	NA	NA	Survived
Thomas, 2007	Tennessee	M/44	Kidney	*E. chaffeensis*	4 y	Fever, epigastric pain, nausea, cough	PCR	NA	NA	NA	Survived
Thomas, 2007	Tennessee	M/63	Heart	Undetermined *Ehrlichia* species	11.6 y	Fever, nausea, vomiting, diarrhea	PCR	NA	NA	NA	Survived
Thomas, 2007	Tennessee	M/36	Kidney	*E. chaffeensis*	2.6 y	Fever, headache	PCR	NA	NA	NA	Survived
Thomas, 2007	Tennessee	M/61	Heart	*E. chaffeensis*	6.2 y	Fever, headache	PCR	NA	NA	NA	Survived
Thomas, 2007	Tennessee	M/65	Lung	*E. ewingii*	6.1 y	Fever, headache, nausea, vomiting	PCR	NA	NA	NA	Survived
Thomas, 2007	Tennessee	M/51	Kidney	*E. chaffeensis*	1.6 y	Fever, headache, nausea, vomiting	PCR	NA	NA	NA	Survived
Thomas, 2007	Tennessee	M/22	Kidney	*E. chaffeensis*	1.9 y	Fever, diarrhea	PCR	NA	NA	NA	Survived
Thomas, 2007	Tennessee	M/73	Heart	*E. chaffeensis*	14.3 y	Fever	PCR	NA	NA	NA	Survived
Assi, 2007	Wisconsin	M/66	Kidney	*A. phagocytophilum*	9 y	Fever, chills, malaise, diffuse myalgia, and headache	PCR	Y	NA	Post-transplant infection	Survived
Lawrence, 2009	Missouri	F/68	Lung	*E. chaffeensis*	30 mo	Fever, cough	PCR	NA	NA	NA	Survived
Lawrence, 2009	Missouri	F/63	Lung	*E. ewingii*	82 mo	Fever, cough	PCR	NA	NA	NA	Survived
Lawrence, 2009	Missouri	M/57	Lung	*E. chaffeensis*	24 mo	Fever, headache, abdominal pain	PCR	NA	NA	NA	Survived
Lawrence, 2009	Missouri	F/26	Lung	*E. chaffeensis*	126 mo	Fever, headache, nausea, vomiting	PCR	NA	NA	NA	Survived
Lawrence, 2009	Missouri	M/62	Kidney	*E. chaffeensis*	12 mo	Chills, fatigue	PCR	NA	NA	NA	Survived
Lawrence, 2009	Missouri	M/64	Kidney	*E. ewingii*	17 mo	Fever, arthralgias, malaise, weakness	PCR	NA	NA	NA	Survived
Lawrence, 2009	Missouri	M/55	Kidney	*E. chaffeensis*	159 mo	Fever, chills, headache, myalgias	PCR	NA	NA	NA	Survived
Lawrence, 2009	Missouri	M/45	Kidney	*E. chaffeensis*	42 mo	Fever, nausea, vomiting, anorexia	PCR	NA	NA	NA	Survived
Lawrence, 2009	Missouri	M/50	Kidney	*E. ewingii*	101 mo	Fever, rash	PCR	NA	NA	NA	Survived

Author, year†	Recipient state of US residence	Sex/ Age, y	Organ	Genus and species‡	Time, transplant to symptoms	Symptoms/clinical details	Method of diagnosis	Tick exposure	Donor characteristics	Likely source of infection	Recipient outcome
Lawrence, 2009	Missouri	F/65	Kidney	*E. chaffeensis*	97 mo	Fever, headache	PCR	NA	NA	NA	Survived
Lawrence, 2009	Missouri	M/55	Kidney	*E. ewingii*	99 mo	Fever, headache	PCR	NA	NA	NA	Survived
Lawrence, 2009	Missouri	M/48	Kidney	*E. chaffeensis*	84 mo	Fever, headache, nausea, vomiting, diarrhea	PCR	NA	NA	NA	Survived
Lawrence, 2009	Missouri	F/44	Kidney	*E. chaffeensis*	130 mo	Fever, malaise	PCR	NA	NA	NA	Survived
Lawrence, 2009	Missouri	M/34	Kidney	*E. ewingii*	36 mo	Fever, cough, nausea, dyspnea, malaise	PCR	NA	NA	NA	Survived
Lawrence, 2009	Missouri	M/30	Kidney	*E. chaffeensis*	64 mo	Fever, chills, headache, nausea, vomiting	PCR	NA	NA	NA	Survived
Lawrence, 2009	Missouri	F/33	Kidney	*E. chaffeensis*	223 mo	Fever, chills, night sweats, nausea, vomiting	PCR	NA	NA	NA	Survived
Lawrence, 2009	Missouri	F/50	Liver	*E. ewingii*	51 mo	Fever, headache, myalgias	PCR	NA	NA	NA	Survived
Lawrence, 2009	Missouri	M/37	Liver	*E. chaffeensis*	92 mo	Fever, headache, vomiting, lethargy	PCR	NA	NA	NA	Survived
Lawrence, 2009	Missouri	M/58	Liver	*E. chaffeensis*	71 mo	Fever, headache, arthralgias	PCR	NA	NA	NA	Survived
Lawrence, 2009	Missouri	M/51	Liver	*E. ewingii*	65 mo	Fever, headache	PCR	NA	NA	NA	Survived
Lawrence, 2009	Missouri	M/54	Heart	*E. chaffeensis*	109 mo	Fever, headache, nausea	PCR	NA	NA	NA	Survived
Dorn, 2012	Virginia	M/27	Kidney	*E. chaffeensis*	4 y	SIRS, acute cellular rejection, admitted to ICU	Peripheral blood smear, serology	Y, ≤6 wk prior to symptoms	Living	Post-transplant infection	Survived
Kumar, 2014	Kentucky	M/63	Kidney	*E. chaffeensis*	10 y	Fever, leukopenia, thrombocytopenia and elevated transaminases, renal dysfunction, HLH	PCR	NA	Deceased	Post-transplant infection	Survived
Regunath, 2017	Missouri	F/69	Lung	*E. ewingii*	13 y	Fever, chills, nausea, non-bilious vomiting, mild leukopenia and thrombocytopenia	PCR	Y	Deceased, CMV+	Post-transplant infection	Survived
Khatri, 2019	Connecticut	M/67	Kidney	*A. phagocytophilum*	18 y	Malaise, acute diffuse scrotal pain, fever, headache, rhinorrhea, leukopenia and thrombocytopenia	PCR, blood smear	N	NA	NA	Survived
Albitar, 2019	Minnesota	M/33	Liver	*A. phagocytophilum*	17 y	Fever, leukocytosis	PCR, blood smear	Went deer hunting, no mention of ticks	Deceased	Post-transplant infection	Survived

*CMV+, cytomegalovirus positive; HLH, hemophagocytic lymphohistiocytosis; ICU, intensive care unit; NA, not available; SIRS, systemic inflammatory response syndrome
†In addition to these reports, Otrock et al. reported on 51 solid organ transplant and 5 stem cell transplant recipients diagnosed with ehrlichiosis during May 2007–October 2016 at Barnes-Jewish Hospital in St. Louis, Missouri, USA, which were excluded from this review. Characteristics of 56 recipients: median age (range) 56.9y (9–72y); 42M/14F; 37 tick exposure, 19 no tick exposure; 49 infected by *E. chaffeensis*, 7 infected by *E. ewingii*; organs received: 18 kidneys, 12 hearts, 12/56 lungs, 7 livers, 2 kidney and pancreas, and 5 received allogeneic stem cells.
‡Genera: *A., Anaplasma*; *E., Ehrlichia*
